# Changing Views of the Evolution of Immunity

**DOI:** 10.3389/fimmu.2013.00122

**Published:** 2013-05-21

**Authors:** Larry J. Dishaw, Gary W. Litman

**Affiliations:** ^1^Division of Molecular Genetics, Department of Pediatrics, University of South FloridaTampa, FL, USA

Views on the evolution of immunity have been redefined as studies in protostomes, invertebrate deuterostomes, and various vertebrates have elucidated molecular details of host defense (Litman and Cooper, 2007; Boehm, 2012). Diverse species possess vast repertoires of immune related defenses, which have evolved into sophisticated, integrated networks (Rast and Messier-Solek, 2008; Messier-Solek et al., 2010). Certain components of immune defense represent either homologous structures or reflect novel approaches to confronting pathogens and other environmental influences [e.g., VLRs in agnathans (Boehm et al., 2012)]. Disparate diversification mechanisms and exceptional sophistication in immune mediators in some species blur distinctions between innate and adaptive immunity, the latter of which is viewed traditionally as a vertebrate adaptation (Litman et al., 2005; Messier-Solek et al., 2010; Boehm, 2011) associated with extensive somatic diversity, antigen-specific affinity maturation, and memory (Flajnik and Kasahara, 2010; Boehm, 2012). Innate immunity, specifically the recognition of microbe-associated molecular patterns by pattern recognition receptors (PRRs), has taken center stage owing to its capacity to shape adaptive immunity (Schenten and Medzhitov, 2011). PRRs [e.g., toll-like receptors (TLRs)], also contribute significantly to immune homeostasis (Medzhitov, 2010; Carvalho et al., 2012). In this special issue we explore topics that are continuing to reshape our interpretations of immune evolution.

Historically, transplantation immunology framed our understanding of immunological recognition and the interplay between immunoglobulin domain-containing receptors, co-receptors, and the major histocompatibility complex (MHC) (Brent, 2003). These earlier concepts were extended to address graft rejection in jawless vertebrates as well as select invertebrates (Finstad and Good, 1964; Hildemann and Thoenes, 1969; Mayer et al., 2002; Little et al., 2005; Kvell et al., 2007). Today, various models of allorecognition are recognized, some of which are restricted to certain phyla (Buss, 1987), and can be traced to the ancestors of sessile invertebrates (Dishaw and Litman, 2009). Broad rules govern discrimination between conspecifics (Rosengarten and Nicotra, 2011). Nydam and De Tomaso (2011) update our understanding of the evolution of allorecognition, emphasizing commonality in the systems that generate polymorphisms, and discuss how genetic diversity is maintained.

Extensive variation in immune genes traditionally has been equated with the immunoglobulin and T cell receptor gene loci in B and T lymphocytes, respectively, as well as in some MHC loci (Hughes, 2002). Recent studies in some invertebrate deuterostomes provide evidence for expansion and germline diversification of immune receptor repertoires. Buckley and Rast (2012) demonstrate lineage-specific properties among expanded sea urchin TLRs. Their findings indicate that: (1) some antigen binding sites may be co-evolving with variable ligands, (2) TLR subfamilies are utilized differently between larval and adult coelomocytes, and (3) sea urchin TLRs most likely represent immune surveillance molecules. Satake and Sekiguchi (2012) review the evolution and functional diversification of TLRs among deuterostomes, highlighting a reduced repertoire in the tunicate, *Ciona intestinalis*. Only two TLRs can be detected in this species, with presumed hybrid functionality *in vitro* (Sasaki et al., 2009). Interestingly, neither TLR1 nor TLR2 recognizes bacterial lipopolysaccharide (LPS), suggesting that *Ciona* utilizes other mechanisms to detect LPS or that an accessory molecule(s) is involved.

*Drosophila melanogaster* (fruit fly) uses complex alternative RNA splicing to diversify the Down's syndrome cell adhesion molecule (DSCAM), a multiexonic receptor implicated in neuronal patterning (Shi and Lee, 2012). Some DSCAM isoforms serve as PRRs in peripheral hemocytes and exhibit increased specificity for distinct targets (Watson et al., 2005; Brites et al., 2008; Chou et al., 2009). These findings are reminiscent of the fibrinogen-related proteins (FREPs) (Adema et al., 1997; Zhang et al., 2004), which consist of fibrinogen and immunoglobulin superfamily-related domains that can undergo somatic mutation and gene conversion. Individual somatic lineages expressing FREPs respond to specific parasite burdens (Mone et al., 2010). Smith (2012) reviews Sp185/333 genes, a large family of innate receptors in sea urchin expressed in hemocytes. Variation in genes encoding Sp185/333 receptors arises via complex DNA rearrangements and may be influenced by persistent antigenic sources (Buckley et al., 2008; Dheilly et al., 2009).

Not all immune receptors are restricted to foreign determinants (Rabinovich and Croci, 2012). Some glycans can be found on both host and microbial surfaces (Davicino et al., 2011). Vasta et al. (2012) describe an apparent paradox among galectins, which until recently were considered essential in self-recognition (Rabinovich and Croci, 2012). Galectins now are considered PRRs that recognize related glycans on microbes (Sato et al., 2009). PRRs are thought to interact only with microbial products (Kawai and Akira, 2010); some, such as galectins, also may possess discriminatory properties (van Vliet et al., 2008). Galectin self-recognition may require interaction with accessory molecules on self-cells and warrants further investigation.

The role of PRRs in symbiotic relationships likely is ancient (Bosch, 2012), involving complex host-microbial interactions at the surface of mucosal tissues (Duerkop et al., 2009; Round et al., 2011; Wells et al., 2011; Hill et al., 2012). Collins et al. (2012) describe a PRR that may govern such interactions between the bobtail squid and *Vibrio fisheri*, a bacterial symbiont of the light organ. Immune systems appear to have evolved mechanisms that discriminate among symbionts and pathogens, while promoting the former (Speckman et al., 2003; Lee and Mazmanian, 2010; Nyholm and Graf, 2012).

There has been a tendency to oversimplify or even ignore the broader roles of PRRs in host physiology. Arrieta and Finlay (2012) review the complex strategies that are used by gut bacteria to modulate immune homeostasis. The complex roles of adaptive immunity among vertebrates further complicates the roles of PRRs in homeostasis (Lee and Mazmanian, 2010; Hooper et al., 2012). Dishaw et al. (2012) argue that *Ciona intestinalis*, a protochordate, can help define host and microbe interactions at mucosal surfaces. Presumably, rules and relationships that govern homeostasis in this system may help reveal how perturbations can lead to a broad range of intestinal pathologies in higher vertebrates.

Specific molecules have been implicated in intestinal homeostasis and include alkaline phosphatase-intestinal (Alpi), a member of the alkaline phosphatase (Alp) family. One possible role for these molecules is the detoxification of LPS, which in turn minimizes innate responses to commensal or beneficial microbial communities (Beumer et al., 2003; Bates et al., 2007; Lalles, 2010). Yang et al. (2012) describe the complex evolutionary patterns of *Alpi* genes, which appear to be evolving independently in vertebrate, non-vertebrate and insect lineages. All four zebrafish *Alp* genes are shown to be expressed in the intestine, where *alp3* is expressed exclusively. The authors propose that intestinal expression of *Alp* may be an ancestral trait as alkaline-phosphatase-mediated LPS detoxification likely is central to the stability of gut microbe and host interactions.

Phylogenetic considerations, including the use of non-traditional models, have been instrumental in forging new thinking among immunologists (Loker et al., 2004). It is becoming increasingly clear that the immune system may have evolved, not only to recognize potential pathogens but also to help sustain and stabilize beneficial associations at the surface of mucosal tissues. Loker (2012) considers symbiosis as a driver of evolutionary novelty on both sides of the host-parasite struggle. In this broad, topical overview, host immunity is a pervasive requirement and the immune evolutionary process is seen to be influenced by conflict with parasites and/or the need to cooperate with symbionts. The work presented in this series already is proving critical in terms of broadening our view of immune complexity and the multifaceted role of the host-microbe dialog in maintaining homeostasis. Critical departures from our traditional views of immune defense are being revealed in detailed studies of alternative model systems and in turn are reshaping our understanding of immunity in conventional systems.
